# Supplementary material for: Vaccination against Varicella Zoster Virus Infection in Less Developed Regions of Guangdong, China: A Cross-Sectional Serosurveillance Study
Source: Vaccines (Basel). 2023 Feb 21;11(3):494. doi: 10.3390/vaccines11030494 (PMC10056728; doi:10.3390/vaccines11030494)
Supplement: Supplementary file 1 [file vaccines-11-00494-s001.zip › vaccines-2091658-supplementary.pdf]

Supplementary Materials

Table S1. Demographic information of participants.

Age (years)	Zhanjiang			Heyuan			total
	N	male	female	N	male	female	
0	150	0	150	119	61	58	269
＞0~5	131	61	70	120	73	47	251
＞5~10	123	63	60	152	69	83	275
＞10~20	94	42	52	31	25	6	125
＞20~30	465	76	389	33	19	14	498
＞30~40	357	88	269	101	22	79	458
＞40~50	123	44	79	101	30	71	224
＞50~60	187	80	107	98	23	75	285
≥60	1747	809	938	89	44	45	1836
total	3377	1263	2114	844	366	478	4221

Table S2. Seroprevalence of anti-VZV IgG in VarV vaccination participants (1~14 years) in Zhanjiang.

varicella vaccine (dose)	anti-VZV IgG<br>positive<br>no (%)	anti-VZV IgG<br>negative<br>no (%)	p value
0	20 (23.26)	66 (76.74)	
1	49 (31.41)	107 (68.59)	
2	10 (62.50)	6 (37.50)	
Total	79 (30.62)	179 (69.38)	0.007

Table S3. Seroprevalence of anti-VZV IgG in VarV vaccination participants (1~14 years) in Heyuan.

varicella vaccine (dose)	anti-VZV IgG<br>positive<br>no(%)	anti-VZV IgG<br>negative<br>no(%)	p value
0	43 (37.07)	73 (62.93)	
1	56 (40.00)	84 (60.00)	
2	9 (75.00)	3 (25.00)	
Total	108 (40.30)	160 (59.70)	0.038

Table S4. Persistance of anti-VZV IgG in vaccination participants.

varicella vaccine (dose)	Time interval （years）	anti-VZV IgG positive, no(%)	GMC （95%CI, mIU/ml）
0	-	63 (31.19)	795.43（665.29, 925.57）
1	≤1	7 (16.28)	590.32（174.46, 1006.18）
	＞1~3	36 (36.73)	558.61（399.76,717.45）
	＞3~5	38 (37.62)	631.24（419.90, 842.58）
	＞5	24 (44.44)	689.93（507.57, 872.29）
	Total	105（35.47）	616.22 (520.85, 711.60)
2	≤1	13 (65.00)	519.66（347.54, 691.77）
	＞1~3	3 (60.00)	-
	＞3~5	3 (100.00)	-
	Total	19 (67.86)	644.48（433.87, 855.10）

Table S5. Effects of varicella vaccination policy reform.

varicella vaccine (dose)	N	positive no(%)	p value
1a	158	44 (27.85)	
1b	138	42 (30.43)	0.625
2b	28	19 (67.86)	＜0.001
Total	324	105 (32.41)	＜0.001

a/b: The participants received one dose VarV before/after varicella vaccination policy reforming.
